# Sex-Specific Hip Movement Is Correlated With Pelvis and Upper Body Rotation During Running

**DOI:** 10.3389/fbioe.2021.657357

**Published:** 2021-06-21

**Authors:** Maurice Mohr, Robin Pieper, Sina Löffler, Andreas R. Schmidt, Peter A. Federolf

**Affiliations:** Department of Sport Science, University of Innsbruck, Innsbruck, Austria

**Keywords:** running injury, knee pain, gender differences, principal component analysis (PCA), gait retraining, patellofemoral pain (PFP), iliotibial band syndrome

## Abstract

There is a sex bias for common overuse running injuries that are associated with sex-specific hip kinematics. Gait retraining programs aimed at altering hip kinematics may be more efficient if they incorporated an understanding of how hip kinematics are correlated with the movement of the remaining body segments. We applied a principal component analysis to structure the whole-body running kinematics of 23 runners (12 ♀) into k = 12 principal movements (PM_k_), describing correlated patterns of upper and lower body movements. We compared the time-dependent movement amplitudes with respect to each PM_k_ between males and females using a waveform analysis and interpreted our findings according to stick figure animations. The movement amplitudes of two PMs (PM_6_ and PM_8_) showed statistically significant effects of “sex,” which were independent of running speed. According to PM_8_, females showed more hip adduction, which correlated with increased transverse rotation of the pelvis and upper body compared to men. We propose that increased hip adduction and upper body rotation in female runners may be a strategy to compensate for a less efficient arm and upper body swing compared to men. Gait interventions aimed at reducing hip adduction and running-related injuries in female runners should consider instructions for both upper and lower body to maximize training efficacy.

## Introduction

Women and men perform many athletic tasks in a sex-specific manner. One important motivation to study sex-specific movement strategies is a range of musculoskeletal sport injuries, which exhibit a bias such that some injuries are more prevalent in women compared to men and vice versa (Taunton et al., 2002; Emery and Tyreman, 2009; Ristolainen et al., 2009). In running, patellofemoral pain (PFP) and iliotibial band syndrome (ITBS) are among the most common injuries and both injuries are more prevalent in women compared to men (Taunton et al., 2002; Boling et al., 2010).

Many authors have investigated sex-specific running kinematics with the most consistent finding that females show a more adducted hip during the stance phase of running (Ferber et al., 2003; Schache et al., 2003; Chumanov et al., 2008; Phinyomark et al., 2014; Sakaguchi et al., 2014; Willson et al., 2015; Almonroeder and Benson, 2017; Boyer et al., 2017). Traditionally, a more adducted hip is thought to result from sex-specific anthropometrics, especially a greater pelvis width to femoral length ratio in women (Ferber et al., 2003; Chumanov et al., 2008) and has been suggested to contribute to the higher prevalence of PFP in women (Noehren et al., 2007; Neal et al., 2016). Consequently, the goal of several recent interventions in individuals with PFP has been to reduce hip adduction and thereby reduce knee pain (Neal et al., 2016).

While some of these prospective studies have achieved promising pain reductions through hip strengthening and biofeedback-guided gait retraining (Earl and Hoch, 2011; Noehren et al., 2011; Willy et al., 2012), none of the current interventions considers the movement of the upper body during running. Of all cross-sectional studies comparing running kinematics between males and females, only two investigations considered upper body movement and both demonstrated larger oscillations in pelvic and lumbar axial rotation in female compared to male runners (Schache et al., 2003; Bruening et al., 2020). The authors of the most recent report on sex-specific running kinematics speculated that there may be a functional relationship between observed differences in hip, pelvis, and upper body movement but acknowledged that this relationship remains poorly understood (Bruening et al., 2020). A better understanding of sex-specific whole-body movement during running, including the correlation between upper and lower body movement, could enhance the design and effectiveness of gait retraining programs aimed at injury prevention or treatment.

In running, the interaction between lower and upper body motion controls the whole-body angular momentum, particularly in the transverse plane (Hinrichs, 1987; Willwacher et al., 2016). Therefore, we can expect that sex-specific differences in lower extremity movement, such as increased hip adduction in females, are correlated with sex-specific compensatory upper body movements. One powerful approach to investigate whole-body movement patterns is to apply a principal component analysis (PCA) to the marker trajectories resulting from 3D motion analysis (Federolf et al., 2013; Federolf, 2016; Werner et al., 2020). This technique allows to structure the movement into individual movement components, i.e., principal movements (PM), which shed light on patterns of correlated segment movements. The advantages of applying a PCA directly to marker trajectories rather than the more traditional 3D joint angles are (1) that the former does not require assumptions on the orientation of joint axes thus avoiding a potential source of error (Della Croce et al., 2005) and (2) that the movement components dominating each PM can be easily visualized in intuitive stick figure animations (Troje, 2002). Two previous studies successfully applied a kinematic PCA to resolve sex-specific running kinematics but did not use a full-body marker set-up and/or did not report on upper body movement (Maurer et al., 2012; Nigg et al., 2012).

The aim of the current study was to investigate sex differences in whole-body movement patterns during running as quantified by a kinematic PCA. We hypothesized, that sex-specific lower extremity motion, e.g., greater hip adduction in women, would be correlated with sex-specific upper body movements to maintain a balanced and stable gait pattern. The correlation between specific lower and upper body movements would be evident through their joint expression in individual principal movements.

## Materials and Methods

### Study Design and Participants

This was a cross-sectional study to compare whole-body movement patterns between healthy males and females during running. An *a priori* power analysis was conducted based on 10 previously published studies investigating sex-specific movement patterns during running (Malinzak et al., 2001; Ferber et al., 2003; Chumanov et al., 2008; Maurer et al., 2012; Phinyomark et al., 2014, 2015; Sakaguchi et al., 2014; Willson et al., 2015; Almonroeder and Benson, 2017; Boyer et al., 2017). A pooled analysis of these studies yielded an average effect size (Cohen’s d) for kinematic comparisons between males and females of 1.25. With a significance level of ɑ = 0.05 and a desired power = 0.8, the required sample size was calculated as *N* = 24. Exclusion criteria were (1) age outside the range of 18–35 years old, (2) no experience with treadmill running, and (3) lower extremity injuries in the last 6 months prior to study participation. Injuries were defined as events that required medical consultation and/or disruption of sport participation for longer than 2 weeks.

A convenience sample of 24 healthy, physically active adults (12 males, 12 females) volunteered to participate in this study. All participants indicated to be physically active for a duration of at least 1–5 h per week. About 90% (21 out of 24) of participants were physically active at least 5 h per week and dedicated one or more hours to activities involving running. The remaining participants were equally physically active and reported previous running experience but were not involved in a running routine at the time of testing. There were no competitive runners in this sample. Therefore, study participants are considered novice and/or recreational runners (Honert et al., 2020). The data from one male participant had to be excluded due to poor recording quality, yielding a final sample size of *n* = 23. This study was approved by the local ethics board of the University of Innsbruck (Certificate 70/2019) in accordance with the ethical principles of the Helsinki Declaration. Prior to study participation, all individuals provided written informed consent and filled out a Physical Readiness Questionnaire.

### Experimental Protocol

All participants completed a treadmill running protocol while their three-dimensional movement patterns were recorded using an 8-camera Vicon system with a sample rate of 250 Hz (Vicon Motion Systems Ltd., Oxford, United Kingdom). Individuals were equipped with 39 retro-reflective markers according to the Vicon Plug-in Gait full body model. Next, an incremental method was used to determine the participants’ preferred speed (Jordan et al., 2007) with the specific instruction to select a comfortable speed for a 40-min training run. Then, each individual completed a warm-up period consisting of 5 min of brisk walking and 5 min of running at the test speed. Next, we recorded the 3D marker trajectories for 30 s yielding 35–40 full gait cycles per participant, which has been shown to be sufficient for accurately estimating kinematic running patterns (Dingenen et al., 2018; García-Pinillos et al., 2018). Throughout the entire treadmill protocol, participants were wearing a safety harness to avoid the risk of injury in the case of a fall or slip.

### Data Processing and Analysis

#### Marker Trajectories

For this analysis, the marker set was reduced to 30 markers, neglecting all markers that are not symmetric between the left and right sides of the body. The marker trajectories were reconstructed and labeled using Vicon Nexus software (v. 2.9.2). Gap-filling in marker trajectories was performed using Nexus software for small gaps in pelvis and head markers and a validated marker reconstruction technique (Federolf, 2013; Gløersen and Federolf, 2016) based on a PCA for small gaps in other marker trajectories.

#### Principal Movement Analysis

Processed marker trajectories were further analyzed using the publicly available *PMAnalyzer* (Haid et al., 2019). This Matlab-based software implements all steps for conducting a kinematic PCA with the goal to structure the complex whole-body running movement into its main movement components, the PMs (Federolf, 2016).

The PMAnalyzer achieved the following pre-processing steps: (1) Filter marker trajectories using a 4th-order Butterworth low-pass filter with a cut-off frequency at 15 Hz; (2) Build one 7,500 [250 Hz × 30 s] row × 90 marker coordinates matrix for each individual. Each row of these matrices is interpreted as a “posture vector,” containing the posture of a given participant at a given point in time; (3) Subtract the mean posture (mean across rows) from each subject-specific matrix; (4) Normalize the posture vectors of each individual to their mean Euclidean norm to minimize the influence of anthropometric differences between individuals on movement amplitudes (Federolf, 2016); (5) Concatenate all subject-specific matrices row-wise yielding one PCA-input matrix with 172,500 rows [7,500 samples × 23 participants] and 90 columns.

Next, the PMAnalyzer performed a PCA on the input matrix providing a new set of 90 orthogonal base vectors (eigenvectors *v*_*k*_) along with their eigenvalues *ev*_*k*_ to fully describe the running movement. Specifically, each eigenvector represents one principal movement while the corresponding eigenvalue indicates the amount of variance accounted for by this principal movement. The projection of the PCA-input data onto *v*_*k*_ defines the time-dependent principal positions *PP_*k*_(t)*, which quantify the movement amplitudes of a given individual at a given point in time with respect to each principal movement. For this analysis, the number of included *v*_*k*_ were selected such that the corresponding *PP_*k*_(t)* explained 99% of the total movement variance.

#### Gait Cycle Segmentation

The principal positions were segmented and time-normalized according to the instances of foot contact. The time points of right and left foot contact were detected according to the first negative peak in the vertical acceleration profile of the toe marker following a maximum in the pelvis height marking the end of the flight phase. This detection algorithm was modified from a technique described by Schache et al. (2001). In comparison to the toe velocity profile used in Schache et al. (2001) we found the acceleration profile to yield a more consistent detection of ground contact. Time-normalized principal positions for each full gait cycle *i* were computed in three steps: *PP*_*k*_ were resampled to 101 data points for the duration between a right foot strike *FS*_*i,R*_ and the next left foot strike *FS*_*i,L*_. Next, *PP*_*k*_ were resampled to 101 data points for the duration between the current left foot strike *FS*_*i,L*_ and the next right foot strike *FS_*i*__+__1,R_*. Lastly, the two resampled *PP*_*k*_ were concatenated to form one full gait cycle.

#### Principal Positions—Visual Comparison

Subject-average *PP*_*k*_ waveforms for each individual were computed as the mean over the time-normalized *PP*_*k*_ of 34 full gait cycles. Subject-average waveforms were further averaged to create one average male and one average female principal movement pattern (±1SD).

#### Principal Positions—Video Animations

Principal movements were visualized in 2D videos using the PMAnalyzer video function “PM trials/subject.” For one randomly selected participant, this function reconstructs the pattern of correlated 3D marker trajectories for a given principal movement and is computed by multiplying *PP_*k*_(t)* and *v*_*k*_. After reversing the normalization to Euclidean distance and adding the mean posture of the exemplary participant, the principal movement videos can be displayed in the original units of measurement (mm). Due to the smaller movement amplitudes explained by the higher-order principal movements, we amplified the respective *PP_*k*_(t)* with a manually selected amplification factor *AmpFac.* Note that this analysis step is simply for visualizing and interpreting the correlated kinematic patterns described by each PM.

The principal movements, which showed statistical differences between males and females were inspected in further detail. First, one gait cycle of a male and a female average running pattern was reconstructed based on the first 20 *v*_*k*_ and the average, time-normalized *PP_*k*_(t)* of either all males or all females, respectively (sex-specific mean of subject-average *PP_*k*_(t)* waveforms, see section “Principal Positions—Visual Comparison”). The movement in the original coordinate system was derived by reversing the normalization to Euclidean distance (using the average of all norm factors) and adding the mean posture (using the average of all mean postures). For the last two steps, we used grand averages rather than sex-specific averages to isolate differences in the movement pattern from anatomical differences. Second, to visualize the differences in the running movement between males and females explained by one specific principal movement, we amplified the respective *PP_*k*_(t)* with a manually selected amplification factor *AmpFac*. In addition, the created videos were used to generate image sequences illustrating the sex-specific differences in movement patterns at specific time points during the first half of the gait cycle: 5% (right foot early stance), 15% (mid stance), 30% (late stance/push-off), and 45% (early swing).

#### Principal Positions—Waveform Analysis

To investigate quantitatively whether the time-normalized *PP_*k*_(t)* waveforms were different between male and female runners, a second PCA analysis was conducted. Comparing waveforms with the help of a PCA has the advantage that the entire waveform shape and amplitude can be compared between conditions rather than limiting the analysis to discrete time points such as minima or maxima. This waveform-PCA was computed separately for each *PP_*k*_(t)*. As a first step, the time-normalized waveforms of all gait cycles and individuals (34 gait cycles × 23 participants = 782 waveforms) were concatenated into a (782 rows × 201 time points) PCA-input matrix. The second PCA yielded a new set of eigenvectors *w*_*k*_ where the eigenvector *w*_1_ with the highest corresponding eigenvalue *ew*_1_ represented the largest variation in shape and/or amplitude of the analyzed gait cycle waveforms. The projection of the principal position input matrix onto *w*_1_ yielded a score *s*_*i,p*_ for each gait cycle *i* and participant *p*, indicating the extent (positive or negative), to which the analyzed waveform shows the pattern described by *w*_1_. These waveform (*w*_1_) scores *s*_*i,p,k*_ for each *PP_*k*_(t)* were then averaged across gait cycles, yielding one average score per individual and principal movement *s*_*p,k*_, which served as dependent variables for the statistical analyses.

#### Comparison of PM-Based and Joint Angular Representations of Running Kinematics

While the PM-based representation of whole-body movement has been validated to accurately represent the mechanics of the movement system (Federolf, 2016), the research and clinical community is more familiar with the description of running kinematics using 3D joint angles. To provide reference values and illustrate how the PM-based waveform scores relate to a joint angle framework, we investigated the peak-to-peak oscillations (i.e., the joint range of motion, ROM) for selected joint angle profiles. Specifically, we used a full-body musculoskeletal model and a standard inverse kinematics procedure in OpenSim (v. 4.1) to determine the peak-to-peak oscillation in knee flexion/extension and hip adduction/abduction of the left leg during each full gait cycle (Delp et al., 2007; Rajagopal et al., 2016). We selected those joint angles because our analysis yielded two PMs—PM_6_ and PM_8_—that contained sex-specific movement components and visual inspection of those PMs suggested sex-specific knee movement (PM_6_) and hip movement (PM_8_), respectively (see sections “Description of Principal Movements” and “Sex Differences in Running Movement Components”). A more detailed description of the joint angle analysis is provided in the Supplementary Material.

### Statistical Analysis

Descriptive statistics of participant age, height, weight, and running speed were determined according to the sex-specific means and standard deviations. The goal of the primary statistical analysis was to investigate whether the waveform scores *s* corresponding to the shapes of *PP_*k*_(t)* differed between males and females independent of the running speed. Therefore, we performed a set of univariate analyses of covariance (ANCOVA) with “sex” as the independent variable, “running speed” as the covariate, and the scores *s*_*p,k*_ for a specific principal movement *PM*_*k*_ as the dependent variable. Running speed did not differ significantly between males and females according to an independent *t*-test and there was no significant interaction of “sex” and “running speed” on any of the PP scores, justifying the inclusion of running speed as a covariate (Field, 2009). Further assumptions of ANCOVA were confirmed based on a Shapiro–Wilk test (approximate normal distribution of residuals) and a Levene’s test (homogeneity of variances). *Post hoc* tests of the running speed-adjusted scores were conducted to investigate mean differences between males and females. Effect sizes were reported as partial Eta squared.

As described in the “Results” section, *k* = 1,..,12 principal movements were included in this analysis while PM_4_ and PM_5_ were not included in the statistical analysis, yielding m = 10 ANCOVAS. To control for the expected proportion of false discoveries (type I error), we used the Benjamini-Hochberg procedure to adjust our significance level for each ANCOVA according to Eq. 1:

(1) $$\alpha_{a\,d\,j}=\frac{l}{m}\cdot \alpha ,$$

where *l* is the rank of each ANCOVA, ordered according to their *p*-values with respect to the independent variable “sex” (from lowest to highest) (Benjamini and Hochberg, 1995). The same procedure was performed to evaluate the effects of the covariate “running speed.”

In a secondary statistical analysis, we used two general linear regression models to investigate the association between (1) joint ROM in knee flexion/extension and PM_6_ waveform scores (model 1) and (2) joint ROM in hip adduction/abduction and PM_8_ waveform scores (model 2). In both models, the outcome variable was the PM waveform score and the predictor variables included “sex,” the “joint ROM,” and the respective interaction term. Confounding by running speed was assessed but was not present. All assumptions for linear regression (normality of residuals, homogeneity of variance, absence of outliers) were assessed and met. The joint angle analysis was limited to the left leg since the right leg showed nearly identical outcomes.

All statistical analyses were carried out in IBM SPSS Statistics for Windows (v25, IBM Corp., Armonk, NY, United States) at an *a-priori* significance level of α = 0.05.

## Results

### Participant Characteristics

Age, height, mass, and preferred running speed of the male and female participants are presented in Table 1

**TABLE 1:** Participant age, mass, height, and preferred running speed.

	**Female (*n* = 12)**	**Male (*n* = 11)**
Age (years, mean ± SD)	25 ± 4	27 ± 3
Height (cm, mean ± SD)	170 ± 7	181 ± 5*
Mass (kg, mean ± SD)	61 ± 5	75 ± 7*
Preferred running speed (km/h, mean ± SD)	10.1 ± 0.8	10.7 ± 1.0

*Significantly higher compared to females according to an independent *t*-test, alpha = 0.05.

### Description of Principal Movements

The first three principal movements explained ∼90% of the variance contained in the overall running movement. The first 12 principal movements explained 99% of the total variance. The full description of the dominating movement patterns in each PM is summarized in Table 2. PM_1__–__8_ are additionally visualized based on the stick figure animations in the Supplementary Videos 1, 2.

**TABLE 2:**
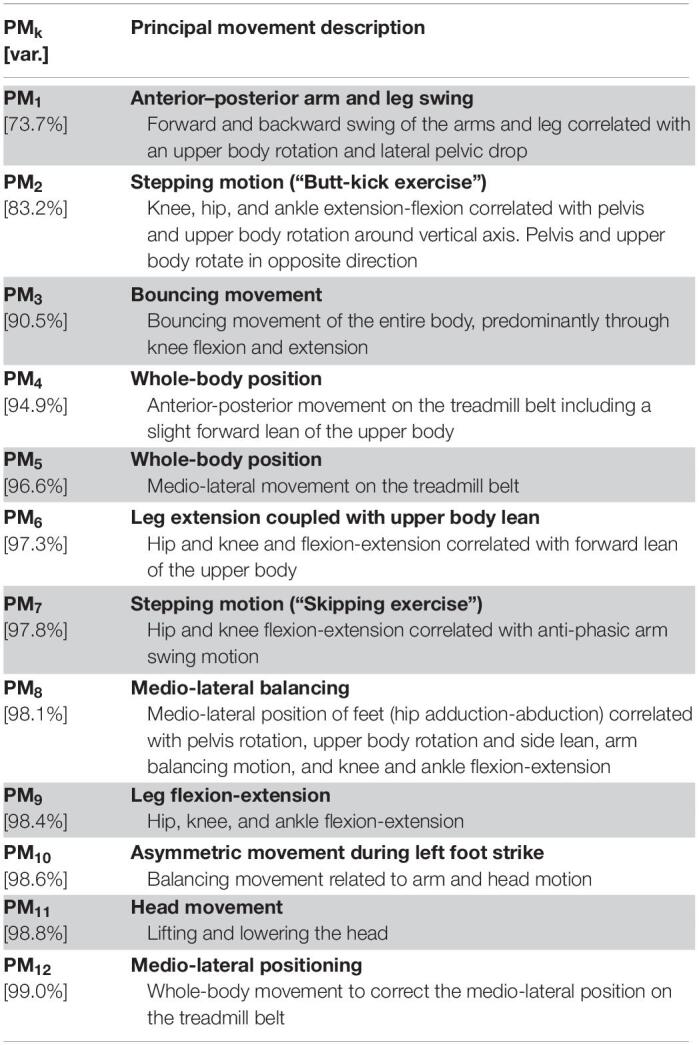
Description of principal movements (PMs) and their cumulative, explained variance relative to the total movement variance (var.).

The first three components explain the main features of the running gait, including the leg swing (PM_1_), the arm swing (PM_1_, PM_2_), corresponding upper body rotation (PM_1_, PM_2_), the stepping motion (PM_2_), and the vertical bouncing motion (PM_3_). PM_4_ and PM_5_ explain the anterior-posterior and medio-lateral whole-body positioning on the treadmill belt. Since the absolute position of the body on the treadmill was not of interest in this study, PM_4_ and PM_5_ were excluded from all further analyses. The functional interpretation of higher-order PMs becomes increasingly difficult since these patterns represent compensatory or complementary balancing movements and postural adjustments, not necessarily visible to the eye when watching a runner. For example, PM_6_ appears to complement PM_2_ and PM_3_ by additionally describing leg extension and upper body forward lean. PM_8_ in contrast seems to represent a medio-lateral balancing strategy including the medio-lateral placement of the feet (i.e., hip adduction-abduction) as well as pelvis and upper body movement in the transverse and frontal planes.

Note that for some PMs, e.g., PM_6_ and PM_7_, the stick figure animations seem to suggest length changes of the thigh and/or shank (Supplementary Video 2). This is a phenomenon created by the fact that the PC vectors form an orthonormal coordinate system for the changes in posture; if rotations of body segments are projected onto only one of the PM-dimensions, then they will appear as length changes of these segments. For comparison, leg or arm swing in gait also appear as segment length changes if observed as a frontal plane projection only. Similarly, segment rotations such as the circular motion path of the feet during running, must be described by the combination of movement along multiple PC vectors with some containing virtual segment deformations that appear unnatural if only one PM is considered (especially after amplification). In general, PMs should not be understood as actual movements but as a coordinate system for the movements of all body segments. Table 2 thus describes the movement aspects that dominate each of the PM_k_ coordinate axes (Federolf, 2016).

### Sex Differences in Running Movement Components

**FIGURE 1:**
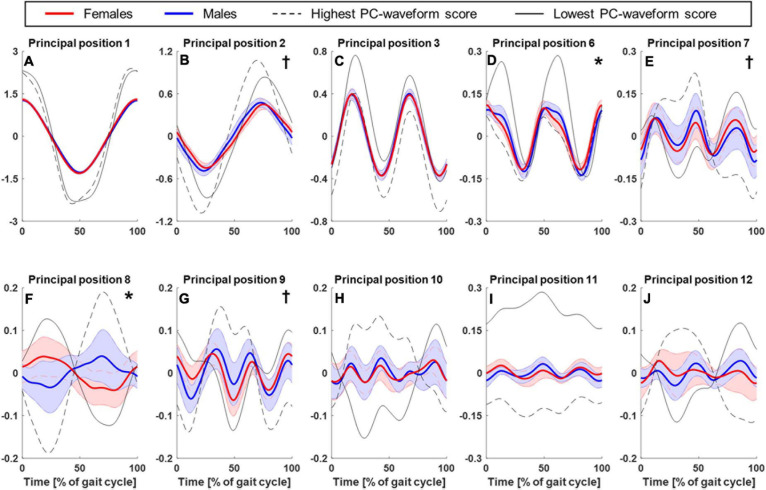
Comparison of principal position waveforms between males and females. Mean and standard deviation (shaded areas) of time-normalized principal position waveforms corresponding to PM1–3 **(A–C)** and 6–12 **(D–J)** for females (red, *n* = 12) and males (blue, *n* = 11). Time point 0% corresponds to a heel strike of the right foot, 50% to a heel strike of the left foot, thus one full gait cycle is shown. Overlaid are those PP-waveforms that scored lowest (solid, black lines) and highest (dashed, black lines) on the first waveform principal components across all participants and gait cycles (see section “Principal Positions—Waveform Analysis”). These waveforms illustrate the main features described by the waveform scores, which were analyzed statistically: “*” and “**†**” indicate a statistically significant effect of “sex” or “running speed” on the principal position scores, respectively.

**FIGURE 2:**
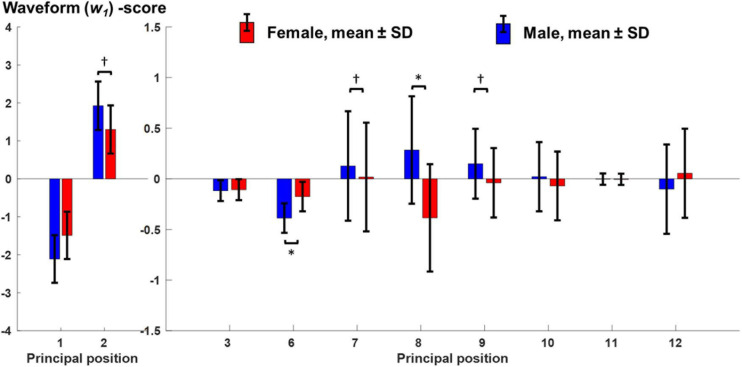
Comparison of PCA-based principal position scores between males and females. Mean and standard deviation of principal position scores regarding waveform PC1 (w_1_) for females (red, *n* = 12) and males (blue, *n* = 11). These values represent the adjusted means with respect to the covariate running speed. “^∗^” and “**†**” mark significant effects of “sex” or “running speed” on the principal position scores after adjusting for multiple comparisons.

Figure 1 shows the sex-specific averages of the time-normalized principal position waveforms for the first 12 PMs (excluding, as discussed earlier, PM_4_ and PM_5_) where the time points 0 and 50% of gait cycle correspond to right and left foot strikes, respectively. Particularly PM_6_ at 10 and 60% of the gait cycle and PM_8_ throughout suggested that males and females showed sex-specific PP(t)-waveforms (Figures 1D,F). The dashed and solid black lines in Figure 1 illustrate the features extracted by the waveform analysis conducted on the principal positions. Specifically, the lines represent the two individual gait cycles that scored lowest and highest on the first waveform principal component (*w*_1_) across all individuals and gait cycles. For example, a high score for PP_8_ resulted from a more male-like waveform shape (start with valley and end with peak) and vice versa for a low score (Figure 1F). Across PMs, Figure 1 demonstrates that the waveform features described by the first waveform principal component coincide with the features that also appear different between males and females. Figure 2 presents the statistical comparison of PP-waveform scores between males and females while considering running speed as a covariate.

#### Medio-Lateral Balancing Strategy (PM_8_)

**FIGURE 3:**
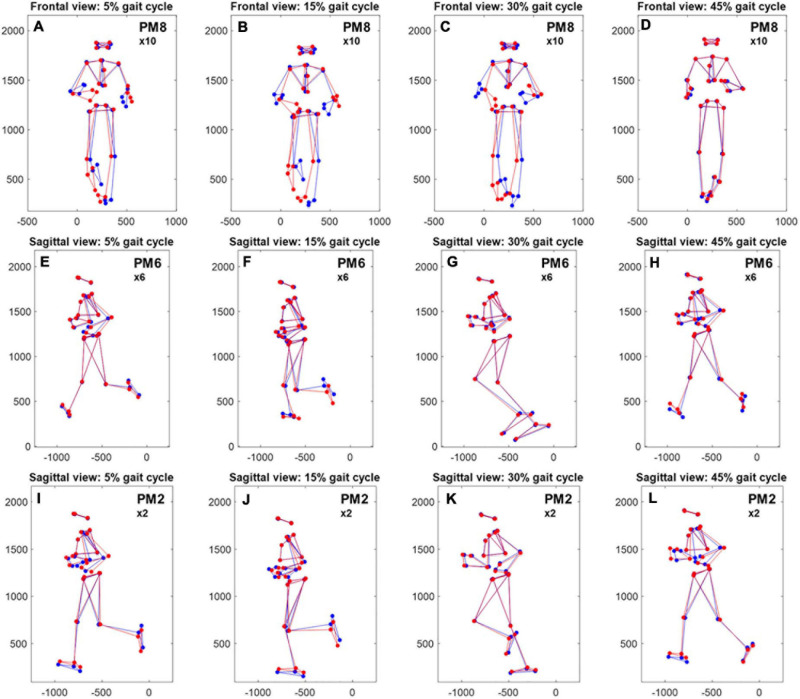
Reconstructed, average movement patterns of males and females. Comparison of reconstructed, average movement patterns of females (red) and males (blue) with amplification of specific principal movements [**(A–D)**, 10 × PM_8_—frontal plane; **(E–H)**, 6 × PM_6_—sagittal plane, **(I–L)**, 2 × PM_2_—sagittal plane]. Reconstructions are based on the first 20 eigenvectors *v*_1__–__20_, the average, time-normalized female and male principal position waveforms (see Figure 1), and the grand average of the mean posture and Euclidean distance normalization factors. x- and y-axes show the distance in mm; the axis labels were removed for better readability.

The most obvious sex-specific difference in principal positions was observed in PM_8_, where males and females showed a mirrored waveform shape. Accordingly, there was a significant, effect of “sex” on PP_8_ scores [*F*(1,20) = 8.54, *p* = 0.008, partial η^2^ = 0.299] without a significant influence of running speed [*F*(1,20) = 0.17, *p* = 0.684, partial η^2^ = 0.008]. Corresponding running-speed adjusted means (±1SD) of PP8-scores were −0.39 ± 0.53 and 0.29 ± 0.53 for females and males, respectively (Figure 2). According to Figures 3A–D and Supplementary video 3 (amplification factor 10), females tended to place their stance foot more medially (indicative of larger hip adduction ROM) while males strike the ground more laterally. At the same time, females showed a larger relative rotation between the pelvis and leg segments and a larger excursion of upper body rotation, both in the transverse plane.

#### Knee Flexion (PM_6_)

A second difference in principal positions was apparent for PM_6_. Specifically, males showed wider PP_6_ maxima (∼10 and 60% of gait cycle) and lower PP_6_ minima (∼30 and 80% of gait cycle). These PP_6_ shape differences were reflected in a significant effect of “sex” on PP_6_ scores [*F*(1,20) = 11.06, *p* = 0.003, partial η^2^ = 0.356]. There was no significant influence of running speed [*F*(1,20) = 1.78, *p* = 0.197, partial η^2^ = 0.082]. Corresponding running-speed adjusted means (±1SD) of PP_6_-scores were −0.18 ± 0.15 and −0.39 ± 0.15 for females and males, respectively. Figures 3E–H and Supplementary Video 4 (amplification factor 5) suggest that this difference originated from variations in knee flexion angle between males and females. Specifically, compared to males, females tended to show a more flexed knee during mid stance (Figures 3E,F) but less knee flexion of the swing leg. In addition, sex-specific variations in the ankle angle around ground contact and push-off were visible in Figures 3E,H.

#### Stepping Motion (PM_2_)

Of the first three PMs, only the second component showed a visibly different shape of the average principal positions between males and females; specifically females showed an average waveform that is shifted to the right and smaller in amplitude (∼25 and 75% of gait cycle). These differences, reflected by PP_2_ scores, were influenced by a significant effect of running speed [*F*(1,20) = 11.65, *p* = 0.003, partial η^2^ = 0.368]. The effect of “sex” on PP_2_ scores [*F*(1,20) = 5.62, *p* = 0.028, partial η^2^ = 0.220] did not reach the adjusted significance level of α_adj_ = 0.015 (*l* = 3 in Eq. 1). Figures 3I–L and Supplementary Video 5 (amplification factor 2) indicate that the observed differences in PP_2_ were mostly related to a difference in swing leg knee flexion. Supplementary Video 5 further suggests that the timing of push-off was affected by the combined influence of running speed and sex.

### The Influence of Speed on Running Movement Components

Further PP scores that showed a significant effect of running speed but no significant sex effect were PP_7_ [speed: *F*(1,20) = 7.82, *p* = 0.011, partial η^2^ = 0.281; sex: *F*(1,20) = 0.22, *p* = 0.646, partial η^2^ = 0.011] and PP_9_ [speed: *F*(1,20) = 10.3, *p* = 0.004, partial η^2^ = 0.340; sex: *F*(1,20) = 1.61, *p* = 0.219, partial η^2^ = 0.075]. PP_1_ scores showed a trend for an effect of both sex [*F*(1,20) = 5.30, *p* = 0.032, partial η^2^ = 0.210] and speed [*F*(1,20) = 4.80, *p* = 0.041, partial η^2^ = 0.193] but did not reach the adjusted significance level.

### Association With Joint Angle Range of Motion

Joint ROM in hip adduction/abduction was a significant predictor of PM_8_ waveform scores (B [95% CI] = −0.07 [−0.10, −0.04], *t*(22) = −4.37, *p* < 0.001) and joint ROM in knee flexion/extension was a significant predictor of PM_6_ waveform scores (B [95% CI] = −0.02 [−0.03, −0.01], *t*(22) = −5.58, *p* < 0.001). Both associations are illustrated in Supplementary Figure 1. In model 2 (PM_8_ vs. hip adduction/abduction), the interaction term with “sex” was a significant predictor of PM_8_ waveform scores, indicating a stronger association in males compared to females (B [95% CI] = 0.08 [0.01, 0.15], *t*(22) = 2.56, *p* = 0.02). A similar interaction was observed in model 1 (PM_6_ vs. knee flexion/extension) but the corresponding coefficient was not statistically significant (B [95% CI] = −0.01 [0.00, 0.02], *t*(22) = 1.99, *p* = 0.06). On average and compared to males, female runners showed a larger ROM in hip adduction/abduction by 7° and a smaller ROM in knee flexion/extension by 6°.

## Discussion

This study tested the hypothesis that the correlated motion of the upper and lower body differs systematically between males and females during running. A kinematic PCA yielded principal movements and corresponding principal positions, i.e., the time-dependent whole-body posture changes associated with each principal movement for either males or females. In support of our hypothesis, we showed distinct visually and statistically significant differences between men and women with respect to the shape and/or amplitude of the principal positions in PM_8_ and PM_6_. For these movement components, there was no evidence for an influence of running speed and they represented a sex-specific balancing strategy including medio-lateral foot placement correlated with pelvis and upper body rotation during stance (PM_8_) as well as sex-specific stance and swing leg kinematics (PM_6_).

Increased hip adduction in female compared to male runners is the most consistent finding related to sex differences in running kinematics (Ferber et al., 2003; Schache et al., 2003; Chumanov et al., 2008; Sakaguchi et al., 2014; Willson et al., 2015; Phinyomark et al., 2016; Almonroeder and Benson, 2017; Boyer et al., 2017). In contrast to the traditional clinical gait analysis, the PCA approach does not express the running movement in terms of 3D joint rotations but creates a new, movement-specific coordinate system where each axis describes correlated movements of body segments. Although conceptually different, the traditional and PCA approach should result in similar observations. The principal positions related to PM_8_ indicated a clear difference in movement strategies between the sexes and the corresponding video animations were indicative of more hip adduction in females, which agrees with previous reports (Ferber et al., 2003; Schache et al., 2003, 2005; Chumanov et al., 2008; Sakaguchi et al., 2014; Willson et al., 2015; Phinyomark et al., 2016; Almonroeder and Benson, 2017; Boyer et al., 2017). Further, our observations confirmed the previous findings that females show more axial rotation of their pelvis (=internal hip rotation) (Ferber et al., 2003; Chumanov et al., 2008; Sakaguchi et al., 2014; Almonroeder and Benson, 2017) and a larger excursion of upper body rotation (Schache et al., 2003; Bruening et al., 2020), although some conflicting evidence exists for these movement features (Malinzak et al., 2001; Maurer et al., 2012; Phinyomark et al., 2014). The regression analysis partially validated our interpretation of the PM animations, revealing that a larger ROM in hip adduction/abduction predicts smaller PP_8_ waveform scores and smaller ROM in knee flexion/extension predicts higher PP_6_ waveform scores (i.e., a more female-like pattern). Currently, we are unsure why these associations were stronger in male runners compared to female runners. Given that our waveform analysis considered only the scores of the first waveform principal component (explaining about 50% of the waveform variance depending on the PM), we speculate that some additional information related to female runners may be contained in higher waveform principal components that were not considered.

The new information, that the current study provides is that sex-specific hip movement is inherently linked to pelvis and upper body rotation, which confirms an assumption of previous investigators (Noehren et al., 2007; Bruening et al., 2020). There may be at least three factors to explain a sex-specific whole-body running movement: (1) anthropometrics, (2) muscle strength, or (3) whole-body dynamics, i.e., the interaction of forces and motion across all body segments. There is little evidence, however, for a consistent association between the anthropometrics and kinematics of the pelvis and hip joint during running (Schache et al., 2005; Chumanov et al., 2008). Further, although women do show lower normalized strength of the hip abductors (Claiborne et al., 2006), successful muscle strengthening did not significantly alter hip or pelvic motion in runners (Earl and Hoch, 2011; Willy and Davis, 2011; Neal et al., 2016). These observations suggest that sex-specific whole-body dynamics may be the predominant factor to explain the current findings.

The whole-body angular momentum during running has to be controlled by coordinated movement of leg, pelvis, trunk, arm, and head motion, particularly in the transverse plane (rotation about vertical body axis) (Hinrichs, 1987; Willwacher et al., 2016). During the stance phase, the ground reaction force (GRF) under the stance leg and the concurrent motion of the swing leg generate a transverse moment, the “free moment” that rotates the runner’s pelvis and upper body towards the stance leg (Hinrichs, 1987; Li et al., 2001). Among other factors, the magnitude of the free moment decreases with a more medial placement of the foot (more hip adduction) and/or with a larger opposite angular momentum generated by upper body sway and arm swing (Hinrichs, 1987). Li and colleagues showed that walking with restricted arm motion had a larger influence on the free moment in men compared to women (Li et al., 2001). Furthermore, men show a higher relative upper body muscle mass in comparison to women (Janssen et al., 2000). We thus assume that upper body rotation of female runners may be less effective in balancing the free moment due to narrower shoulders and less mass distributed across arms and upper body (Li et al., 2001). Therefore, women may control the free moment by more hip adduction and a larger upper body swing excursion compared to males. The combination of more hip adduction and pelvis rotation has been shown to put more stress on the patellofemoral joint as well as on the iliotibial band and may thus expose women to a higher risk of injuries of these structures (Ferber et al., 2010; Willy and Davis, 2011). In contrast, males can potentially afford to land with more hip abduction and balance the resulting transverse free moment with more angular momentum of the upper body. Taken together, these findings suggest that gait-retraining interventions with the goal of preventing knee injuries in female runners with excessive hip adduction should include instructions to actively increase arm und upper body swing in order to allow landing with a less adducted hip. Given the correlational evidence provided by the kinematic PCA in this study, however, the proposed relationship should be confirmed in a subsequent study that experimentally manipulates either arm and trunk sway or segment inertial properties and determines the effect on hip movement. Either way, displaying PM-based stick figure animations to the learner to visualize their own as well as a desired movement pattern could provide a powerful tool in a gait retraining setting given the finding that learning is enhanced when individuals try to imitate model movements rather than following instructions related to joint kinematics (Benjaminse et al., 2015).

In contrast to sex-specific hip adduction during running, previous findings for other joints and in other planes of movement are less consistent. For example, some authors have reported that during the running stance phase, females show more knee flexion compared to men (Boyer et al., 2017) while others showed the opposite (Malinzak et al., 2001; Phinyomark et al., 2014), or no significant difference in knee flexion angles (Ferber et al., 2003; Almonroeder and Benson, 2017). Our findings based on PM6 extend those of Boyer et al. (2017) that women tend to bend their knees more during mid-stance. The conflicting findings between investigations of sex-specific leg flexion/extension may arise from variations in study designs with respect to the tested running speeds, i.e., either pre-determined or self-selected speeds. It is known that faster speeds lead to adaptations in the running gait with most pronounced adaptations in basic sagittal plane kinematics (Maurer et al., 2012). This is reflected in our analysis, showing a significant influence of running speed on PM_2_ principal positions and a trend of an effect of speed on PM_1_ principal positions. These movement components mainly describe leg and arm motion in the sagittal plane. If male and female movements are compared at a constant speed, e.g., 3.5 m/s, the relative speed (with respect to their maximum) is likely faster for females compared to males, which could confound the kinematic comparison. On the other hand, if running kinematics are compared at a self-selected pace, a lower preferred speed in the female group could equally confound the kinematic analysis. Our solution to this issue was to compare men and women at their preferred running speed and include speed as a covariate in the analysis. This approach allowed us to differentiate between the effects of speed, which are generally present in the basic movement components of running (e.g., PM_2_) (Maurer et al., 2012; Nigg et al., 2012), and the effects of sex, which we identified in PM_6_ and PM_8_. Our findings of more knee flexion during stance in women as well as sex-specific ankle angles at ground contact (see Figures 3F,H) suggest that women may use a modified foot strike pattern from men. This finding further adds to the body of literature concerning sex differences in running kinematics but was not the main focus of this analysis and will not be further discussed.

A limitation of this study is the relatively small sample size when compared to other analyses of sex differences in running kinematics with 100 or more participants (Nigg et al., 2012; Phinyomark et al., 2014). Our literature review, however, resulted in expected large effects and thus justified our sample size. Further, the holistic approach of the kinematic PCA and waveform analysis is likely more sensitive (Federolf et al., 2013) compared to traditional approaches in detecting sex differences in running kinematics since it takes into account all measured marker trajectories as a function of time instead of picking discrete outcome variables. A second limitation is that our sample consisted of physically active, young adults but not necessarily trained runners and thus, our results may not be generalized to other running populations such as elite/experienced or elderly runners. Nevertheless, our findings are applicable to (1) novice runners who are an interesting population due to their relatively high risk of running injury (Nielsen et al., 2012) and (2) recreational runners who form the largest running population (Hoitz et al., 2020). Third, part of our discussion revolves around whole-body rotational moments, which we did not assess directly in this study. Although they should be considered speculative, our arguments appear reasonable given previous evidence of sex differences in free vertical moments during walking, which are closely related to whole-body angular momentum in the transverse plane (Li et al., 2001). Nevertheless, the proposed relationship between a sex-specific body mass distribution, whole-body angular momentum, hip joint movement, and ultimately the risk of running-related injury should be considered a hypothesis to be tested in a future prospective study, which includes a biomechanical model based on sex-specific anthropometrics and segment masses to quantify segment inertial properties. Lastly, the qualitative PM descriptions in Table 2 are interpretations, which are susceptible to observer bias or misinterpretations, marking a limitation of our approach. However, the PMs themselves, including their representation as animations, are objective and purely data-driven outcomes in the current study. By amplifying the influence of individual PMs on the overall movement in Supplementary Videos 1, 2, we attempted to make the movement aspects dominating each PM more obvious. Our approach is strengthened by the result of a significant association between the PM waveform scores and the ROM with respect to those joint angular movements that were evident from the corresponding PM animations.

Based on a whole-body movement analysis, this study confirmed that women demonstrate more hip adduction and pelvis rotation during running compared to men. As a novel finding, we showed that sex-specific hip motion correlates with sex-specific pelvis and upper body movement. Specifically, women use a larger range of motion with respect to pelvis and upper body rotation. We suggest that female runners employ this strategy of hip adduction coupled with more upper body rotation to maintain a whole-body angular momentum of close to zero in the transverse plane, potentially compensating for a less efficient arm swing and trunk sway. The correlation between lower body and upper body mechanics should be considered when designing interventions aimed at reducing hip adduction and internal rotation as risk factors for overuse injuries during running.

## Data Availability Statement

The datasets presented in this study can be found in online repositories. The names of the repository/repositories and accession number(s) can be found below: Mohr et al., 2021.

## Ethics Statement

The studies involving human participants were reviewed and approved by local ethics board of the University of Innsbruck (Certificate 70/2019). The patients/participants provided their written informed consent to participate in this study.

## Author Contributions

MM, RP, SL, and AS conducted pilot tests and recruited participants. RP, SL, and AS carried out measurements. MM, RP, and SL processed the marker trajectories. MM analyzed the data and wrote the first manuscript draft. All authors conceived the study and its design, revised the manuscript, and read and approved the final manuscript version.

## Conflict of Interest

The authors declare that the research was conducted in the absence of any commercial or financial relationships that could be construed as a potential conflict of interest.
