# Supplementary material for: Sex-Specific Hip Movement Is Correlated With Pelvis and Upper Body Rotation During Running
Source: Front Bioeng Biotechnol. 2021 Jun 21;9:657357. doi: 10.3389/fbioe.2021.657357 (PMC8255915; doi:10.3389/fbioe.2021.657357)
Supplement: Supplementary file 6 [file Data_Sheet_1.docx]

Supplementary Material

All supplementary materials are accessible via Mendeley: <http://dx.doi.org/10.17632/ndvj69k7bx.2>

# Supplementary Video Content

Supplementary Video File S1 - Visualizations - PM1-4.mp4

Supplementary Video File S2 - Visualizations - PM5-8.mp4

Supplementary Video File S3 - Female vs. male movement - 10xPM8.mp4

Supplementary Video File S4 - Female vs. male movement - 6xPM6.mp4

Supplementary Video File S5 - Female vs. male movement - 2xPM2.mp4

Note: For some PMs, e.g. PM6 and PM7 supplementary video file S2 seems to suggest that these PMs represent length changes of the thigh and/or shank. This is a phenomenon created by the fact that the PC vectors form an orthonormal coordinate system for the changes in posture; if rotations of body segments are projected onto only one of the PM-dimensions, then they will appear as length changes of these segments. For comparison, leg or arm swing in gait also appears as segment length changes if observed only as a frontal plane projection. Similarly, segment rotation such as the circular motion path of the feet during running, has to be described by the combination of movement along multiple PC vectors with some containing virtual segment deformations that appear unnatural if only one PM is considered (especially after amplification).

# Supplementary Description of Joint Angle Analysis

## Methods of joint angle analysis

We used a full-body musculoskeletal model and a standard inverse kinematics procedure in OpenSim (v. 4.1) to determine the peak-to-peak oscillation (i.e. range of motion (ROM)) in knee flexion/extension and hip adduction/abduction of the left leg during each full gait cycle (Delp et al., 2007; Rajagopal et al., 2016). The model has been described in detail by Rajagopal and colleagues (2016) and consisted of 22 articulating bodies including 20 and 17 degrees of freedom for the lower and upper body, respectively. Scaling of the generic model to a subject-specific model was performed using the OpenSim scaling tool based on a neutral upright standing trial (one second long) that each participant had completed at the beginning of the measurement session. For the scaling procedure, the highest scaling weights were assigned to markers that can be placed on anatomical landmarks with high reliability such as the markers on the pelvis, lateral malleoli, lateral knees, elbows, wrists, acromia, and torso. The inverse kinematic procedure was performed on the same window of running marker data that had been used for the kinematic PCA described in the main article. Following the joint angle analysis, the hip adduction/abduction and knee flexion/extension waveforms for each participant were exported from OpenSim and imported into the custom-written Matlab script for further processing. Joint angle waveforms were filtered using a 4th-order Butterworth low-pass filter with a cut-off frequency at 15 Hz. Based on the detected time points of left foot contact (see manuscript section 2.3.3 ‘Gait cycle segmentation’), we determined the joint ROM in knee flexion/extension and hip adduction/abduction as the angular displacement between the respective minimum and maximum joint angle for each full gait cycle. We then derived one average joint ROM outcome variable per participant and joint movement (knee flexion/extension and hip abduction/adduction).

## Results of joint angle analysis

On average (± SD), female and male runners showed a joint ROM of 79.7° ± 10.0° and 88.8° ± 5.6° knee flexion/extension and a joint ROM of 26.5° ± 6.1° and 19.3° ± 4.5° in hip abduction/adduction, respectively. An average difference of seven degrees in hip abduction/adduction ROM is in good agreement with Schache and colleagues (2003) who reported an average difference of 6.6 degrees.

The association between the joint ROM outcome variables and the PP waveform scores for PM_6_ and PM_8_ (see manuscript section 2.3.6 and 2.3.7) are shown in Figure S1. Please note that the joint ROM variables have been centered around zero.


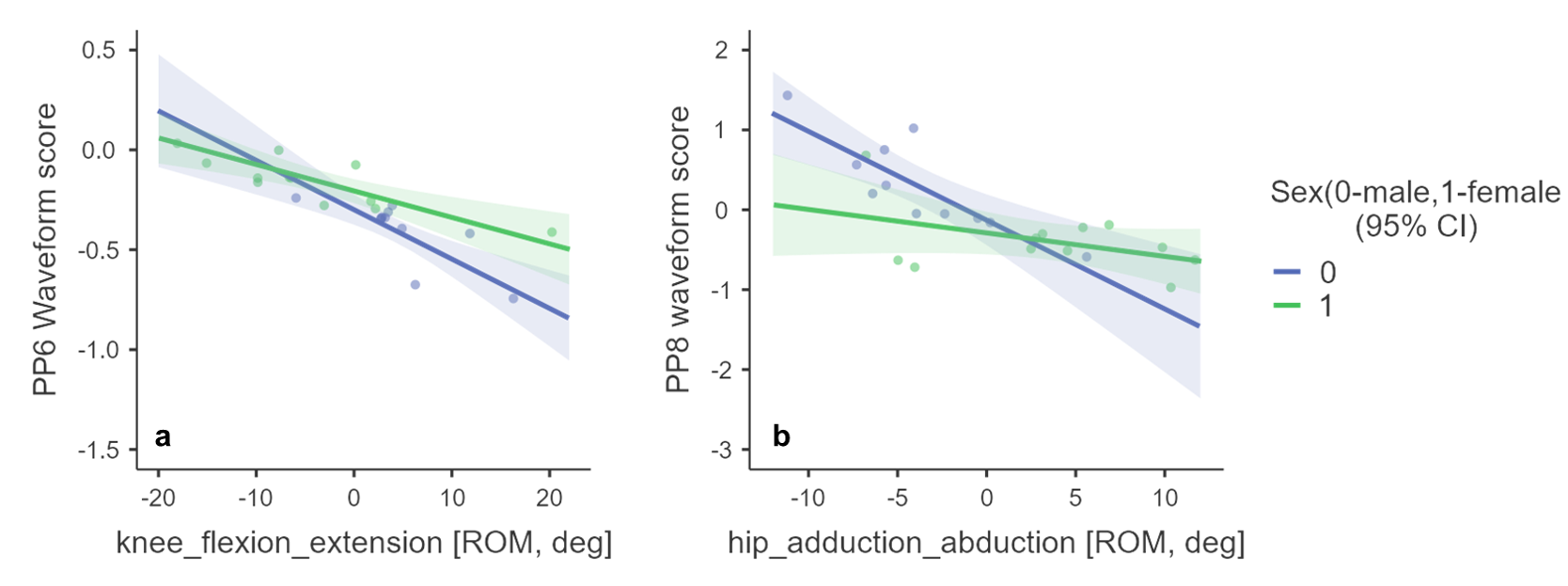


**Supplementary Figure 1.** Association between joint angle ROM and PM waveform scores. Association between knee flexion/extension ROM and PP_6_ waveform scores (a) and between hip adduction/abduction ROM and PP_8_ waveform scores (b) for females (green dots and lines) and males (blue dots and lines). Shaded areas illustrate the 95% prediction confidence intervals.

# References

Delp, S. L., Anderson, F. C., Arnold, A. S., Loan, P., Habib, A., John, C. T., et al. (2007). OpenSim: Open-Source Software to Create and Analyze Dynamic Simulations of Movement. *IEEE Transactions on Biomedical Engineering* 54, 1940–1950.

Rajagopal, A., Dembia, C., DeMers, M., Delp, D., Hicks, J., and Delp, S. (2016). Full body musculoskeletal model for muscle-driven simulation of human gait. *IEEE Transactions on Biomedical Engineering*, 1–1.
